# Intestinal fluid absorption in anadromous salmonids: importance of tight junctions and aquaporins

**DOI:** 10.3389/fphys.2012.00388

**Published:** 2012-09-28

**Authors:** Kristina S. Sundell, Henrik Sundh

**Affiliations:** Fish Endocrinology Laboratory, Department of Biology and Environmental Sciences, University of GothenburgGothenburg, Sweden

**Keywords:** intestinal fluid absorption, aquaporin, claudin, paracellular permeability, salmonids, Ussing chambers, osmoregulation

## Abstract

The anadromous salmonid life cycle includes both fresh water (FW) and seawater (SW) stages. The parr-smolt transformation (smoltification) pre-adapt the fish to SW while still in FW. The osmoregulatory organs change their mode of action from a role of preventing water inflow in FW, to absorb ions to replace water lost by osmosis in SW. During smoltification, the drinking rate increases, in the intestine the ion and fluid transport increases and is further elevated after SW entry. In SW, the intestine absorbs ions to create an inwardly directed water flow which is accomplished by increased Na^+^, K^+^-ATPase (NKA) activity in the basolateral membrane, driving ion absorption via ion channels and/or co-transporters. This review will aim at discussing the expression patterns of the ion transporting proteins involved in intestinal fluid absorption in the FW stage, during smoltification and after SW entry. Of equal importance for intestinal fluid absorption as the active absorption of ions is the permeability of the epithelium to ions and water. During the smoltification the increase in NKA activity and water uptake in SW is accompanied by decreased paracellular permeability suggesting a redirection of the fluid movement from a paracellular route in FW, to a transcellular route in SW. Increased transcellular fluid absorption could be achieved by incorporation of aquaporins (AQPs) into the enterocyte membranes and/or by a change in fatty acid profile of the enterocyte lipid bilayer. An increased incorporation of unsaturated fatty acids into the membrane phospholipids will increase water permeability by enhancing the fluidity of the membrane. A second aim of the present review is therefore to discuss the presence and regulation of expression of AQPs in the enterocyte membrane as well as to discuss the profile of fatty acids present in the membrane phospholipids during different stages of the salmonid lifecycle.

## Introduction

Anadromous salmonids are born in fresh water (FW), migrate to the sea to forage and grow before becoming sexually mature and return to their natal rivers to spawn (McCormick et al., 1998). Thus, the typical life cycle of anadromous salmonids includes two important transitions between FW and seawater (SW). The current overview will focus on the first osmotic transitional stage, i.e., the pre-adaptation of the hyperosmoregulatory parr in FW to become a hypoosmoregulatory smolt in SW.

The anadromous salmonids show a wide spectrum of complex changes in physiology, morphology, biochemistry, and behavior that take place in FW, during the parr-smolt transformation, pre-adapting the fish for a life in SW. The developmental changes are governed by a number of endocrine systems, of which cortisol is a major component together with growth hormone (GH), insulin-like growth factor-I (IGF-I), and thyroid hormones (McCormick et al., 1998).

**Figure 1:**
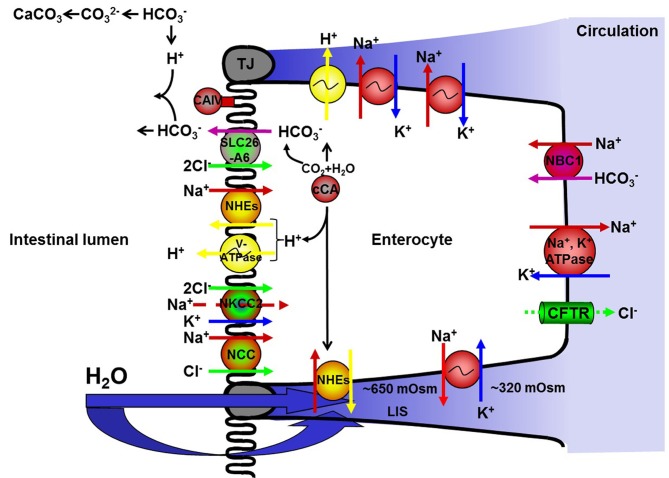
**A simplified version of the current hypothesis regarding ion-driven intestinal fluid absorption in stenohaline marine and seawater acclimated euryhaline teleosts (see text for details).** NKA; Na^+^, K^+^-ATPase, NKCC; Na^+^:K^+^:2Cl^−^ co-transporter, NCC; Na^+^:Cl^−^ co-transporter, NHEs; Na^+^/H^+^ exchangers, CAc; cytosolic carbonic anhydrase, tCAIV; membrane bound carbonic anhydrase type IV, V-ATPase; V-type H^+^-ATPase, Na^+^:HCO^−^_3_ co-transporters; NBCs, Cl^−^/HCO^−^_3_ exchanger; SLC26A6. CFTR; cystic fibrosis transmembrane conductance regulator.

In teleost fish, continuously exposed to osmotic forces across all epithelia, transitions between environmental salinities requires marked changes in osmoregulatory mechanisms in order to maintain osmotic homeostasis. In FW, active absorption of ions and excretion of the excess amounts of water diffusing into the fish is crucial. In SW, on the other hand, uptake of water in combination with secretion of ions is needed (see Marshall and Grosell, 2005; Evans, 2008). For both passive and active movements of ions and water, all epithelia: intestine, gills, kidney, and skin, are involved, but the main epithelia responsible for a regulated fluid intake is the intestine. A prerequisite to perform this task is to have access to ingested water. Already in 1930, Smith demonstrated that eel (*Anguilla anguilla*) in SW had higher drinking rates compared to FW conspecifics. High drinking rates have since been described as a general feature for several stenohaline marine and euryhaline SW acclimated species when compared to fish living in FW (Perrott et al., 1992). However, water cannot be actively absorbed and therefore the water uptake was suggested to be coupled to an uptake of monovalent ions (Smith, 1930). For stenohaline SW fish, solute linked water absorption has been extensively investigated and molecular mechanisms including active monovalent ion transporters, ion-channels and co-transporters have been presented (Figure 1; see Grosell, 2011 for details). Several of the active transport mechanisms suggested for stenohaline SW fish are present also in anadromous salmonids but the detailed characterization and localization of all various transporters are not yet performed. Furthermore, not only the transporting function of the solutes determines the intestinal fluid uptake, but equally important for an efficient absorption of fluid across the intestinal epithelium are the characteristics and regulation of the intestinal epithelial permeability. Present models for intestinal fluid uptake in stenohaline teleosts do not reveal the relative importance of paracellular versus transcellular pathways or the components responsible for allowing the movement of water across the epithelia. In this respect the intestine of anadromous salmonids, which changes physiological mechanisms to meet FW and SW environments, respectively, can provide a valuable model system. The salmonid intestine offers a powerful and general model to study regulatory mechanisms of and pathways for water movement during intestinal fluid absorption also beyond the boundaries of teleost osmoregulation.

## Methodological considerations

In order to study intestinal fluid uptake in salmonids, several methods have been used. The classical everted and non-everted gut sac preparations have been used to measure intestinal fluid uptake in fish from different external salinities as well as in different regions of the gastrointestinal tract (Collie and Bern, 1982; Usher et al., 1991; Veillette et al., 1993; Cornell et al., 1994; Kerstetter and White, 1994; Veillette et al., 1995; Madsen et al., 2011). To assess the contribution of active ion transporters to the intestinal fluid uptake, the main focus has been directed toward the intestinal Na^+^, K^+^-ATPase (NKA) activity, using NKA enzyme specific *in vitro* assays (Colin et al., 1985; Madsen, 1990; Bisbal and Specker, 1991; Seidelin et al., 2000; Stefansson et al., 2003; Sundell et al., 2003; Veillette and Young, 2004; Veillette et al., 2005). The recent expansion in knowledge regarding the genomes of multiple salmonid species has led to a rapid increase in development of both molecular and immunological tools. These tools have opened up for the assessment of specific target genes for several other transporters also involved in the ion coupled fluid transport, i.e., NKA, Na^+^/H^+^ exchangers (NHEs), cytosolic carbonic anhydrase (CAc), membrane bound carbonic anhydrase type IV (tCAIV), V-type H^+^-ATPase (V-ATPase), Na^+^:HCO^−^_3_ co-transporters (NBCs), FXYDs, claudins, aquaporins (AQPs), Na^+^:K^+^:2Cl^−^ co-transporters (NKCCs), Na^+^:Cl^−^ co-transporter (NCC) and Cl^−^/HCO^−^_3_ exchanger (SLC26A6) (Grosell et al., 2007, 2009; Tipsmark and Madsen, 2007; Tipsmark, 2008; Tipsmark et al., 2008; 2010a,b; Madsen et al., 2011) as well as immunostaining of specific target proteins (AQPs, CAc, tCAIV, V-ATPase) which has been important to determine the abundance and localization of these ion transporters (Seidelin et al., 2000; Grosell et al., 2007; Madsen et al., 2011).

In order to get an overall picture of the entire intestinal epithelia and the physiological mechanisms involved in fluid transport including the barrier creating proteins, live epithelia can be studied, *in vitro*, using the Ussing chamber methodology (Ussing and Zerahn, 1951). This is an established technique for measurements of active and passive transports and transfer of solutes and water across a live epithelial tissue. The Ussing chambers were first described by the Danish physiologist Hans H. Ussing, who studied the capacity of an epithelium to actively move ions and nutrients against an electrochemical and/or concentration gradient using the frog skin as a model (Ussing and Zerahn, 1951). Today, modified Ussing chambers, or diffusion chambers (Grass and Sweetana, 1988), are widely used to study epithelial physiology in a multitude of species and tissues for applications such as ion transport, nutrient uptake, protein absorption, drug absorption, host pathogen interactions, and pathophysiological mechanisms (Santos et al., 2000, 2001; Saunders et al., 2002; Sundell et al., 2003; Velin et al., 2004, Jutfelt et al., 2006, 2007, 2008; Moeser et al., 2007; Clarke, 2009). Within the pharmacuetical sciences and industry, diffusion chambers are routinely used for high troughput screening of substance absorption across epithelia. This has led to a simplification of the classical Ussing chamber technique by exclusion of one or both electrode set ups, a clear disadvantage when investigating detailed physiological processes within epithelia. One of the great advantages with the Ussing-type of diffusion chambers is the two electrode pairs equipping the chambers and measuring the electrical characteristics of the epithelium. These electrical characteristics will provide information on both preparation viability and valuable information on transporting activities and permeability. Each chamber is equipped with one pair of KCl-electrodes to measure the potential difference across the epithelium and one pair of inert (e.g., platinum) electrodes that can be used to apply currents or voltages. The measuring electrodes are continuously monitoring the transepithelial potential (TEP) and in the original Ussing chamber set up, increasing currents were applied across the epithelium until a TEP of zero was reached. That current was named the short circuit current (SCC) and is equivalent to the sum of the ion movements induced by active transport. The transepithelial resistance (TER) could then be calculated from the TEP and the SCC, using Ohms' law (Ussing and Zerahn, 1951). However, the classical Ussing method has been further developed with time, and one main improvement of the methodology is the use of alternating small currents after which the resulting voltages are recorded, at time intervals, instead of applying a larger current clamping the epithelium. This way, electrical charging of the epithelium is prevented and more undisturbed electrical measures possible (see Wikman-Larhed and Artursson, 1995 and Sundell et al., 2003). Our laboratory has further refined this methodology by the development of a new Ussing chamber measurement system: UCC-401 (UCC-Labs Ltd.) that applies alternating adaptive DC voltages (U) to the epithelium generating corresponding currents (I). The voltages are randomly applied to generate currents that alternate between positive and negative values, within the range of a fixed min and max value, resulting in zero net charge. The range of currents is manually defined, i.e., between −30 and 30 μA and the currents are generated during four consecutive cycles to generate mean values. The DC voltages are applied through the use of platinum electrodes, every 5th minute, to minimize electrical loading of the epithelium. The U/I pairs obtained are fitted to a straight line using the least square method. The slope of the line represents the TER and the voltage where it intercepts *U* = 0, show the SCC. Undisturbed TEP is continuously measured using the pair of KCl electrodes immersed in 3 M KCl and connected to a beaker with 0.9% NaCl using KCl-agar bridges (4% agar in 3 M KCl) from the NaCl beaker a second set of agar bridges made in 0.9% NaCl connect to the Ussing chambers as close as possible to the epithelium (Sundell et al., 2003).

Using the Ussing chamber methodology, the TEP obtained is a result of the whole epithelium and will thus be a sum of ion transfer both through the paracellular pathway and via the electromotive forces of the basolateral and apical membrane in series (Halm et al., 1985a). A serosa-negative potential would reflect a net uptake of negative charges and most likely a diffusion of positive ions (Na^+^) back to the lumen through possible cation-selective tight junctions (TJ), whereas a serosa-positive potential would reflect a net uptake of positive charges and may indicate a lower permeability for cations across TJ. The SCC across the epithelium reflects an overall net ion transport activity and includes apart from the major ions, Na^+^ and Cl^−^, also K^+^ and HCO^−^_3_.

The TER equals the sum of the paracellular shunt resistance and the transcellular resistance, in which the transcellular resistance is determined by the apical and the basolateral membrane resistances in series. The fish intestine is mostly defined as a leaky epithelium (Powell, 1981; Loretz, 1995), and thus the TER is regarded to reflect the paracellular permeability, i.e., the conductance across the TJ but can under certain conditions also be influenced by the lateral intercellular space (LIS) (Blikslager et al., 2007). However, this assumption is dependent on the relative contribution of the para- and transcellular resistances to the TER, which differs between regions of the gastrointestinal tract as well as between the species and environmental conditions (Sundell et al., 2003; Jutfelt et al., 2006, 2008; Sundh et al., 2010). The permeability of the paracellular pathway can additionally be studied by the use of hydrophilic permeability markers such as ^14^C-mannitol, which is a hydrophilic molecule, suggested to be passing only through the paracellular pathway (Bjarnason et al., 1995). Urea, a smaller but equally uncharged and hydrophilic molecule, has also been used as a paracellular marker (Artursson et al., 1993). However, urea may also have a transcellular uptake route through specific urea transporters. In fish, the specific urea transporter (UT-b) is present in kidneys and gills (Walsh et al., 2000; Mistry et al., 2005), but has to our knowledge not yet been demonstrated in the intestine. Therefore urea may still be a relevant paracellular marker in this epithelium (Artursson et al., 1993). On the other hand, the UT-b transporter belongs to the same family as the Na^+^-coupled glucose transporter (SGLT1) (Leung et al., 2000) which is an important transporter in the intestinal epithelium and interactions may be possible making the route of urea transfer a bit more unpredictable also in the intestine.

## Salmonids and intestinal ion transport

**Figure 2:**
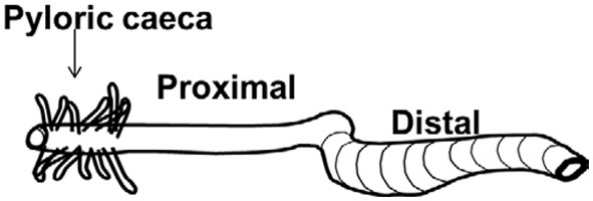
**A schematic drawing of the gastrointestinal tract of salmonid fish.** In this review the intestinal region just after the pyloric caeca is termed as the proximal intestine. The region just behind the ileo-rectal valve is termed the distal intestine.

As shown for stenohaline SW fish, salmonids in SW display elevated drinking rates (Usher et al., 1988; Fuentes et al., 1996) in association with elevated intestinal fluid absorption (Collie and Bern, 1982; Veillette et al., 1993, 2005; Kerstetter and White, 1994; Nielsen et al., 1999; Genz et al., 2011; Madsen et al., 2011). The major driving force and hence the first step in intestinal fluid transport (Figure 1) in salmonids is considered to be basolateral located NKA (Loretz, 1995; Veillette et al., 2005; Madsen et al., 2011). The selective NKA inhibitor, ouabain, decreases the Jv across intestinal sac preparations by 67–100% in coho salmon (*Oncorhynchus kisutch*; Collie and Bern, 1982), Atlantic salmon (*Salmon salar* L.; Veillette et al., 1993) and rainbow trout (*Oncorhynchus mykiss*; Madsen et al., 2011) and the elevated fluid absorption in SW is associated with elevated NKA activities throughout the whole intestinal canal (see Figure 2 for description of the intestinal regions), from the pyloric caeca (Rey et al., 1991; Seidelin et al., 2000; Veillette et al., 2005) to the proximal (Colin et al., 1985; Veillette et al., 1995; Sundell et al., 2003) and distal intestine (Colin et al., 1985; Sundell et al., 2003).

During smoltification, the intestine, as well as the other osmoregulatory tissues, will pre-adapt for a life in SW, while the fish are still in FW. A developmental increase in drinking rates (Nielsen et al., 1999) and increased fluid absorption have been observed in the distal intestine at the peak of smoltification in Atlantic salmon (Veillette et al., 1993) and brown trout (*Salmo trutta* L.) (Nielsen et al., 1999), whereas the proximal intestine does not seem to show the same increase (Veillette et al., 1993; Nielsen et al., 1999). Increased fluid absorption at the peak of smoltification is accompanied by increased NKA activity, which in Atlantic salmon is apparent in both proximal and distal intestine (Sundell et al., 2003).

The second step in the solute coupled fluid absorption (Figure 1) is the intake of Cl^−^ ions into the enterocytes. In marine teleosts, this is mainly governed by an apically situated NKCC2 or for some species by a NCC, both driven by the inwardly directed Na^+^-gradient (Field et al., 1978; Frizzell et al., 1979, 1984; Musch et al., 1982; Halm et al., 1985b; Tresguerres et al., 2010; Watanabe et al., 2011). The presence and expression profile of NKCC2 or NCC in Atlantic salmon intestines during smoltification and SW acclimation is poorly investigated but is under assessment in our lab. In salmonids, Cl^−^ absorption in exchange for HCO^−^_3_ (SLC26A6) has received increased attention the last decade following its first demonstration in rainbow trout (Shehadeh and Gordon, 1969). This exchange is suggested to contribute to the solute coupled fluid absorption both by intake of Cl^−^ by the enterocytes and by reducing the luminal osmolality through an increases supply of HCO^−^_3_ for precipitation of luminal Ca^2+^ into CaCO_3_ (see Grosell, 2010, 2011). Several intracellular sources for HCO^−^_3_ have been suggested, including a basolateral NBC1 as well as intracellular CO_2_ hydration facilitated by CAc (Grosell et al., 2007). Hydration of CO_2_ generates a surplus of H^+^ which have to be excreted to avoid cellular acidification and a basolateral as well an apical V-ATPase together with NHEs has been suggested in rainbow trout acclimated to SW (Grosell et al., 2007). As far as we know, the expression profile and significance of NBC, SLC26A6, and CAc during smoltification and SW acclimation of Atlantic salmon intestine, is mainly unexplored and certainly needs attention.

## The permeability of the salmonid intestinal epithelium—implications for fluid absorption

While the major mechanisms for the ion transport in SW fish, i.e., the driving force behind the fluid transport across the intestine is thoroughly investigated, the main route for water flow, is under debate and has not yet been established (Alves et al., 1999; Hill et al., 2004; Fischbarg, 2010; Laforenza, 2012). The permeability for both transcellular and paracellular routes can be physiologically regulated. The paracellular permeability is mainly regulated by affecting the TJs (Madara and Pappenheimer, 1987; Daugherty and Mrsny, 1999; Anderson et al., 2004), whereas the transcellular permeability to water can be regulated by the composition of the polar membrane lipids (Haines, 1994; Hill et al., 1999) and/or by incorporation of AQPs into the membranes (Ma and Verkman, 1999; Nedvetsky et al., 2009; Laforenza, 2012). Thus, depending on the relative permeability of these two pathways the fluid absorption could be shunted between the paracellular and the transcellular route. In this aspect the anadromous salmonids are very interesting to study as they migrate between a hypoosmotic and hyperosmotic environment also including dramatic changes in degree of exposure of the environment to the intestinal epithelium.

### Paracellular permeability at different environmental salinities and during smoltification

**Figure 3:**
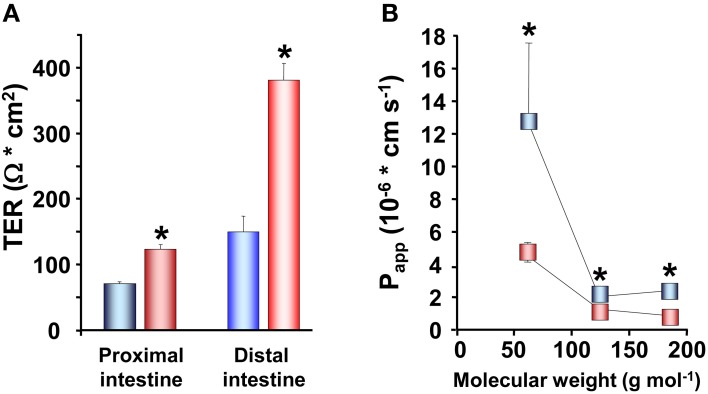
**Transepithelial resistance (TER; *n* = 8) in the proximal and distal intestine (A) and the apparent permeability coefficient (P_app_; *n* = 16) (B) for three different sized hydrophilic permeability markers; ^14^C-urea, MW:62, ^14^C-erytritol, MW:124 and ^14^C-mannitol, MW:184, in the proximal and distal intestine of fresh water** (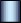, 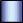) **and seawater** (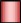, 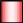) **adapted rainbow trout**. (Values presented are means ± SEM). Asterisks indicate significant differences between FW- and SW-acclimated trout. All data are expressed as mean values ± SEM. Differences in the parameters measured are analyzed using Two-Way ANOVA followed by Student Neuman Keuls *post-hoc* procedure. Significance was accepted at *p* < 0.05 (see Sundell et al., 2003; Jutfelt et al., 2007; Sundh et al., 2009).

In studies on rainbow trout, the electrical characteristics of the intestinal epithelium have been investigated at different salinities. Rainbow trout, provided by Anten fish farm (Alingsås, Sweden), were of both sexes (body weight 100–150 g; *n* = 88) and maintained in re-circulated, filtered, and aerated FW for at least 10 days after transfer to the laboratory. Subsamples of fish were transferred to SW (25‰) and allowed to acclimate for at least 3 weeks to the new environment. Intestines of fish maintained in FW as well as acclimated to SW were sampled and mounted in Ussing chambers as described by Sundell et al. (2003). The TER, TEP, and SCC were monitored together with radiotracer studies assessing the apparent permeability (P_app_) of three different sized hydrophilic markers: ^14^C-urea; MW:62, ^14^C-erytritol; MW:124 and ^14^C-mannitol; MW:184. SW acclimated trout showed higher TER than the FW acclimated, in all intestinal regions examined (Figure 3A). This was confirmed by measurements of P_app_ for the three hydrophilic marker molecules. P_app_ was lower for all three markers in SW than in FW acclimated trout and followed a trend where the P_app_ decreased with increasing molecular size in all cases (Figure 3B). This suggests that decreased intestinal paracellular permeability is a hallmark for salmonids after acclimation to SW. In agreement, increased TER in both proximal and distal intestine and reduced P_app_ for mannitol after acclimation to SW have been observed in several studies on Atlantic salmon (Sundell et al., 2003; Sundh, Nielsen, Stefansson, and Sundell, in preparation; Sundh, Nielsen, Stefansson, Andersson, Taranger, and Sundell, in preparation; Sundh, Nielsen, Andersson, Taranger, Schultz, Prunet, Stefansson, and Sundell, in preparation). Considering the importance of an osmotic gradient in the LIS which creates the driving force for fluid transport (see Grosell, 2010, 2011), the decrease in the permeability of the paracellular pathway after migration to SW may be advantageous to the fish. By reducing conductance for ions through TJs when the fish are in SW, this would increase the ability of the fish to build up the osmotic gradient in the LIS. Moreover, a higher TER and considerably lower P_app_ for all paracellular marker molecules were observed in the rainbow trout distal compared to the proximal intestine (Figures 3A,B). This is normally observed also in Atlantic salmon, making this regional differentiation a common feature for these two salmonids (Sundell et al., 2003; Jutfelt et al., 2006, 2008; Sundh et al., 2010). Since the distal intestine appears to be the dominating water absorbing region in salmonids in SW, this suggests that the higher TER in this region is of functional importance for fluid absorption, probably by decreasing the leakage of especially positive ions back into the intestinal lumen. This is however, somewhat contradictory to one earlier study on Coho salmon (Collie, 1985) where no change in TER was seen in the proximal intestine whereas a decrease was seen in posterior intestine after SW acclimation.

**Figure 4:**
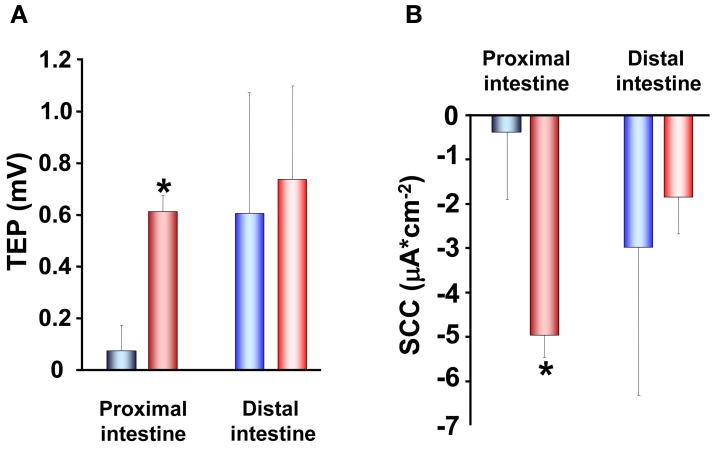
**Transepithelial potential (TEP; A) and short-circuit current (SCC; B) in proximal and distal regions of the intestinal tract of fresh water** (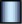, 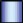) **and seawater** (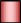, 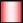) **adapted rainbow trout (*n* = 8)**. Asterisks indicate significant differences between FW- and SW-acclimated trout. All data are expressed as mean values ± SEM. Differences in the parameters measured are analyzed using Two-Way ANOVA of variance, followed by Student Neuman Keuls *post hoc* procedure. Significance was accepted at *p* < 0.05 (see Sundell et al., 2003; Jutfelt et al., 2007; Sundh et al., 2009).

The increase in TER observed in SW acclimated trout is also reflected in the other electrical parameters. Proximal intestines from SW acclimated fish show a more serosa positive TEP than intestines from FW acclimated trout (Figure 4A). This is an expected consequence of the increase in TER and can be explained by reduced conductance and thus a reduction in the cation leakage from the hyperosmotic LIS to the intestinal lumen. Further, there were higher absolute values in SCC of SW acclimated trout compared to FW acclimated trout (Figure 4B). No difference was observed between FW and SW acclimated trout in the distal intestinal region, which can be a result of the distal region being less prone to have gradient building active transport mechanisms as this region do not actively absorb nutrients to a significant extent (Loretz, 1995; Bakke-McKellep et al., 2000; Jutfelt et al., 2007). The serosa positive TEP in SW seems to be quite specific for salmonids (Figure 4A; (Oxley et al., 2007; Sundh et al., 2010, 2011), as when comparing with electrical data from more stenohaline SW fish, most previous literature show serosa negative TEP (see Loretz, 1995). However, this is not a totally universal pattern, since small serosa-positive potentials have been reported also for one marine teleost *Cottus scorpius* (House and Green, 1965). In FW acclimated fish, on the other hand, the TEP is often more close to zero and can fluctuate between serosa positive and serosa negative values even within the same species (Figure 4A; Sundh, Nielsen, Andersson, Taranger, Schultz, Prunet, Stefansson, and Sundell, in preparation; Huang and Holt, 1974; Ando, 1975; Ando et al., 1975). The combined pattern of responses in electrical parameters after acclimation of FW trout to SW, supports each other and suggests a model in which the ion transporting activities increases (as seen by a higher absolute value for the SCC in SW) in order to desalt the ingested SW and thereby reducing the luminal osmolality to allow for fluid uptake. Concomitantly the paracellular permeability decreases to allow for building up a sufficient osmotic gradient in the LIS, and thus creates a strong driving force for fluid absorption.

Another important aspect of a tighter intestinal epithelium, i.e., increased TER in SW acclimated fish, is an increased physical barrier function. This would favor the disease resistance, as SW is a thriving habitat for both bacteria and viruses (Wilhelm and Suttle, 1999) and with increasing drinking rates in SW the load of potentially harmful substances will increase. Hence, a second advantage of decreased paracellular permeability in SW would be to reduce the ability for antigens and other harmful substances to get access to the host via the paracellular pathway thus increasing the disease resistance by creating a stronger intestinal barrier (Sundh et al., 2009; Ahrne and Johansson Hagslätt, 2011; Segner et al., 2012).

### Claudin expression at different salinities and during smoltification—the link to paracellular permeability

The TJ consists of several physiologically regulated proteins forming the circumferential seals around adjacent epithelial cells. Three of the main protein families found in the TJs are occludins, claudins, and junction-associated membrane proteins (JAM). The claudins and occludins form the backbone of the TJ, and the number of TJ strands is suggested to be proportional to the permeability (Schneeberger and Lynch, 2004; Van Itallie et al., 2008). However, the selective permeability to different both charged and uncharged molecules are much more complex and discrepancies between the measures of the paracellular pathway when using the electrical parameter TER or the apparent molecular permeability of a hydrophilic molecule, P_app_, are not infrequent (Sundell et al., 2003; Van Itallie et al., 2008; Sundh et al., 2010, 2011). One probable explanation for this is that TER is measured within milliseconds, whereas P_app_ is measured as fluxes over hours and therefore also represents the dynamics of the TJs. TJs have been shown to frequently break, migrate and reconnect which would allow for alternating movement of molecules over time (Anderson et al., 2004; Van Itallie and Anderson, 2006). Claudins, as well as occludin and other adjacent proteins affect the selective permeability for different molecules, and this selectivity is not only dependent on molecular size but also on electrical charge (Van Itallie et al., 2008; Vikström et al., 2009; Cummins, 2012).

The claudins constitute a large protein family, with several different isoforms in fish. In the pufferfish (*Fugu rubripes*), 56 different claudin isoforms are described (Loh et al., 2004) and the number of isoforms presently known in Atlantic salmon are 26 (Tipsmark et al., 2008). The different claudin isoforms display different number and types of charged amino acid residues lining the pore that is formed between the adjacent cells which constitute the passage way for molecules using the paracellular pathway. The differential expression of claudin isoforms has therefore been suggested to be the main determiners of TJ ion and size selectivity (Van Itallie and Anderson, 2006; Amasheh et al., 2011). This suggestion is based on detailed functional studies in mammals, whereas in fish, the knowledge on the physiological characteristics of the different claudin isoforms is limited. As far as we know, one study in zebrafish (*Danio rerio*) has suggested claudin-15 to be a cation pore forming isoform (Bagnat et al., 2007) and another study has demonstrated that claudin-30 reduces sodium permeability in the gills of Atlantic salmon (Engelund et al., 2012). In mammals, claudin-1, -3, -4, -5, -8, -11, -14, and -19 are all described as barrier builders, claudin-2 and -10 are suggested to create cation selective pores, whereas the role for claudin-7, -12, -15, and -16 is still obscure (Amasheh et al., 2011). Interestingly, claudin-2 appears, in addition to increase the cation permeability, also to create a paracellular water channel through the TJ (Musch et al., 2010). In the Atlantic salmon intestine, recent work has revealed the mRNA expression of several claudin isoforms. These includes claudin-3a, -3b, -3c, claudin-15, and claudin-25b of which the two latter are suggested as isoforms specific for the intestine (Tipsmark et al., 2008, 2010a; Tipsmark and Madsen, 2012). Based on comparison of the charged amino acid residues in the part of claudin sequences located within the paracellular space between adjacent enterocytes, claudin-15 in Atlantic salmon was suggested to be similar to zebrafish and mammalian claudin-15, a pore forming isoform, while Atlantic salmon claudin-25b displayed similarities with mammalian claudin-4, a barrier building claudin (Tipsmark et al., 2010a). Moreover, two other proteins that are known to be important in the formation of TJs are occludin and tricellulin and the presence of these in the salmonid intestine have been shown at the mRNA level (Tipsmark and Madsen, 2012). Thus, these proteins are also believed to be important players in determining the structure and function of the TJ complex.

Baring the nature of the claudins in mind, an increased TER after SW transfer could thus be the result of an increased expression of barrier builders and/or a reduced expression of pore forming claudins. Throughout the whole intestinal length, the mRNA levels of claudin-25b was >10 times more abundant compared to claudin-15 and claudin-3 (Tipsmark et al., 2010a; Tipsmark and Madsen, 2012) suggesting that claudin-25b is the dominating claudin in the intestine of Atlantic salmon. Moreover, the expression of claudin-25b was 10–20 times higher in the distal region compared to the proximal region which would support the suggestion that claudin-25b is, similarly to mammalian claudin-4, a barrier building claudin, as the permeability is normally lower in distal compared to the proximal intestine (Sundell et al., 2003; Jutfelt et al., 2006, 2008; Sundh et al., 2010; Tipsmark et al., 2010a). Altogether, this suggests that claudin-25b is one important determiner of the character of TJ properties and that changes in this isoform determine/dominate the physiological effects observed in epithelial permeability measured as TER. Thus, the higher TER normally observed in the distal intestine compared to the proximal intestine (Figures 3A and 6A; Sundell et al., 2003; Jutfelt et al., 2006, 2008; Sundh et al., 2010) could be explained by the higher expression of claudin-25b in this intestinal region. Further, the increased expression of claudin-25b in the proximal intestine of SW acclimated Atlantic salmon (Tipsmark et al., 2010a) clearly supports and also provides an explanation to the increase in TER seen in SW exposed salmonids (Figure 2A; Sundell et al., 2003; Sundh, Nielsen, Stefansson, and Sundell, in preparation).

### Cortisol as a regulator of intestinal epithelial transport and paracellular permeability

**Figure 5:**
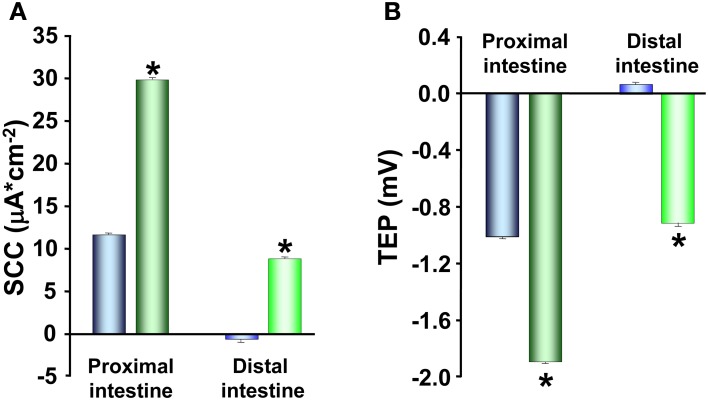
**Short-circuit current (SCC; A) and transepithelial potential (TEP; B) in proximal and distal intestine of control** (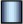, 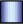) **and cortisol** (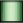, 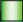) **implanted rainbow trout acclimated to fresh water**. Values presented are means ± SEM (*n* = 8). Asterisks indicate significant differences between cortisol treated and control trout, in each region of the intestinal tract. All data are expressed as mean values ± SEM. Differences in the parameters measured are analyzed using Two-Way ANOVA of variance, followed by Student Neuman Keuls *post hoc* procedure. Significance was accepted at *p* < 0.05 (see Sundell et al., 2003; Jutfelt et al., 2007; Sundh et al., 2009).

Cortisol has a major developmental role in the smoltification of salmonids and the increase in plasma levels during this life stage is well established (Specker, 1982; Specker and Schreck, 1982; Young, 1986; Shrimpton and McCormick, 1998; Sundell et al., 2003). *In vivo* injections of cortisol stimulate NKA activity in the intestine of rainbow trout (Madsen, 1990) as well as increases intestinal fluid absorption of several salmonid species (Cornell et al., 1994; Specker et al., 1994; Veillette et al., 1995, 2005). Both *in vivo* and *in vitro* treatment with cortisol stimulates NKA activity and fluid absorption in the pyloric caeca of Chinook salmon (*Oncorhynchus tshawytscha*; Veillette and Young, 2004; Veillette et al., 2005). Moreover, administration of the corticosteroid antagonist RU486 abolished the increased fluid absorption observed during natural smoltification as well as after *in vivo* treatment with slow release cortisol implants (Veillette et al., 1995). Thus, the intestinal fluid transport seems to be fully regulated by cortisol and in order to provide a more detailed view on the mechanisms by which cortisol act to increase the intestinal fluid uptake, Ussing chambers have been used to study electrophysiology of rainbow trout intestines after treatment with slow release implant of cortisol as described by Specker et al. (1994). The cortisol implant procedure has been shown to chronically elevate plasma cortisol concentrations in a physiological range over 7 days and significantly stimulate intestinal fluid uptake in post-smolt stage Atlantic salmon 5–7 days after administration of the implant (Cornell et al., 1994; Specker et al., 1994; Veillette et al., 1995). After 7 days of cortisol treatment the SCC showed a higher absolute value with similar effect for both intestinal regions (Figure 5A). This suggest that cortisol stimulates the epithelial ion transporting activity, which is in line with previously shown cortisol stimulation of fluid transport, increases in NKA activity in salmonids during smoltification as well as the concomitant increase in NKA activity and plasma cortisol levels after SW transfer (Colin et al., 1985; Rey et al., 1991; Veillette et al., 1995, 2005; Seidelin et al., 2000; Sundell et al., 2003). A stimulation of SCC was also observed when FW acclimated trout was acclimated to SW, which presents further support for the role of cortisol as a main regulator of the increased ion-transporting capacity occurring during parr-smolt transformation.

**Figure 6:**
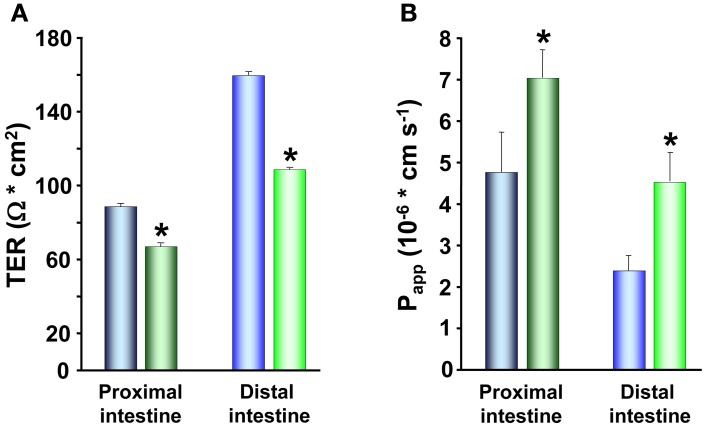
**Transepithelial resistance (TER; A) and the apparent permeability coefficients (P_app_; B) of ^14^C-mannitol, MW:184, in proximal and distal intestine of control** (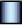, 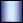) **and cortisol** (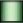, 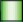) **implanted rainbow trout acclimated to fresh water**. Values presented are means ± SEM (*n* = 8). Asterisks indicate significant differences between cortisol treated and control trout, in each region of the intestinal tract. All data are expressed as mean values ± SEM. Differences in the parameters measured are analyzed using Two-Way ANOVA of variance, followed by Student Neuman Keuls *post hoc* procedure. Significance was accepted at *p* < 0.05 (see Sundell et al., 2003; Jutfelt et al., 2007; Sundh et al., 2009).

The electrical parameter reflecting intestinal permeability, TER, as well as the P_app_ both demonstrated increased paracellular permeability in response to cortisol treatment. The proximal and distal intestine showed decreased TER (Figure 6A) and increased P_app_ for mannitol (Figure 6B) in the cortisol treated trout compared to controls, suggesting that cortisol modulates the composition of proteins in the TJ complex. Indeed, Atlantic salmon receiving injections with cortisol downregulate claudin-25b, the probable barrier building claudin, in the proximal intestine of FW acclimated fish and in both proximal and distal intestine of SW acclimated fish (Tipsmark et al., 2010a). Thus, a reduction in claudin-25b may be an explanation behind the increased paracellular permeability seen in the cortisol treated rainbow trout. Moreover, a reduction in TER can be observed close to the peak of smoltification (Sundell et al., 2003; Sundh, Nielsen, Stefansson, and Sundell, in preparation) when the plasma cortisol levels are peaking. In agreement, mRNA levels of claudin-15 and -25b decrease during smoltification with lowest levels just prior to SW transfer (Tipsmark et al., 2010a), suggesting that cortisol might be responsible for these developmental changes seen in paracellular permeability.

When implanting rainbow trout with cortisol, TEP show a more serosa negative value (Figure 5B) which in parallel to the findings in paracellular permeability is contradictory to the response observed after transfer to SW (Figures 3 and 4; Sundell et al., 2003). Taken together, cortisol seems to be the main regulatory hormone of the increased intestinal fluid uptake necessary for salmonids when transferred to SW. This is achieved through a stimulation of enterocyte NKA activity during smoltification as well as after SW transfer. However, cortisol treatment of rainbow trout and increasing circulating levels of cortisol in Atlantic salmon during smoltification instead result in increased paracellular permeability, which is counter to the pattern seen after SW transfer. A possible explanation to these discrepancies is that cortisol stimulates the active transporting activities during smoltification, while the fish are still in FW, thus equipping the intestine with the right set of transporting proteins for a SW environment. The effects of cortisol on the paracellular permeability on the other hand, is to remain a high or even increased permeability, and the increase in serosa negative TEP suggests that this increased paracellular permeability is cation selective. A leakage of positive ions back to the intestinal lumen would thereby prevent the buildup of an osmotic gradient in the LIS and thus allow for a preparatory increase of ion transporting activities without creating a too high fluid absorption while the fish is still in FW.

## Transcellular permeability at different salinities and during smoltification

In salmonids, during smoltification as well as during cortisol treatment, the paracellular permeability is maintained high and any fluid absorption would probably mainly occur through a paracellular route. However, after SW transfer there is a decrease in the paracellular permeability of the epithelia together with an increased NKA activity of the basolateral enterocyte membrane. This clearly suggests a re-direction of the water flow from a paracellular route in FW to a more transcellular route in SW, anticipating increased transcellular permeability for water in SW. An increase in the water permeability of the enterocyte membrane could be due to altered permeability of the lipid bilayer or to incorporation and/or activation of AQPs.

### The role of the lipid bilayer

The enterocyte membrane, being an epithelial cell with major transporting functions, contains a large fraction of transport proteins, but also, a large area of lipid bilayer. In the enterocyte plasmamembrane phospholipids play a major role for fluidity and permeability (Stubbs and Smith, 1984; Seo et al., 2006) and changes in the composition of fatty acids in this cell membrane may thus have a major impact on the transcellular water permeability. Since the gastrointestinal tract is the first organ to encounter ingested feed, the lipid composition of the fish diet has shown to influence the lipid composition of the enterocyte membrane (Houpe et al., 1997; Cahu et al., 2000; Ruyter et al., 2006). Moreover, it is clear that also the external environment have an impact on enterocyte membrane composition as the fatty acid profiles can change after SW acclimation even though the same diet is maintained. Transfer of masu salmon (*Oncorhynchus masou*) and rainbow trout from FW to SW resulted in an increased level of *n*−3 poly unsaturated fatty acids (*n*−3 PUFA) of the intestinal brush border membrane (Leray et al., 1984) and total intestinal tissue (Li and Yamada, 1992). This increased proportion of *n*−3 PUFA in the brush border membrane was concomitant with an increased fluidity of the membrane (Leray et al., 1984). Alteration of PUFA incorporation into cell membranes is a physiological control mechanism to alter fluidity of the membranes in response to changes in temperature and hydrostatic pressure. Regarding the intestinal epithelial membranes this increase is suggested to result in increased water permeability (Brasitus et al., 1986; Lande et al., 1995). However, our most recent and preliminary results using NMR diffusometry of lipid vesicles prepared from intestinal mucosa of Atlantic salmon reared in FW or SW, show no major differences in water permeability (Bernin, Claesson, Sundh, Olsen, Andersson, Nydén, and Sundell, in preparation). This suggests that the protein part of the cell membranes have a larger influence than the lipid bilayer, on the transcellular water permeability. The most plausible explanation for an increased protein mediated transcelluar fluid absorption would be through incorporation of AQPs. This has elegantly been shown to be the physiological regulation of water transport in other transporting epithelia, like the classical trafficking model of AQP2 in mammalian kidney cells (Nedvetsky et al., 2009).

### The role of AQPs

The existence of cellular water channels was heavily disputed until evidence was presented in 1992 (Preston et al., 1992). AQPs are divided in two subfamilies; orthodox AQPs transporting only water and aquaglyceroporins that in addition, transport solutes like glycerol. In the human genome, 13 AQPs has been identified (AQP0-12) and an intense period of research have followed the AQP discovery showing the importance of AQPs in, e.g., absorption of water in the kidney, balance of the osmotic pressure in the brain tissue, tumor growth, and oocyte maturation (Verkman, 2009, 2012). Also, the importance of AQPs in the gastrointestinal tract of mammals has been highlighted (Ma and Verkman, 1999; Matsuzaki et al., 2004; Laforenza, 2012). Much less is knowm about AQPs in fish but data on the importance of AQPs in the gastrointestinal tract is growing (see Cutler et al., 2007; Cerda and Finn, 2010). However, in order to elucidate the importance of AQPs in salmonids and their role in intestinal fluid absorption during smoltification as well as in different environmental salinities, more studies are essential. The protein abundance and localization of different AQP isoforms at the cellular and sub-cellular levels must be known and supplemented by functional and structural characterization and regulation of the proteins (Walz et al., 2009; Cerda and Finn, 2010).

Recently, initial steps have been undertaken in order to elucidate the role of AQPs in Atlantic salmon during smoltification and after SW transfer. AQP-1aa, -1ab, -8ab and 10 have all been suggested as possible players in transepithelial water transport due to their existence at the mRNA transcript level (Tipsmark et al., 2010b). During Atlantic salmon smoltification, mRNA expression increased for AQP-1aa (pyloric caeca), AQP-8ab (pyloric caeca, proximal, and distal intestine) and AQP-10 (pyloric caeca and distal intestine). After transfer to SW, the expression of these isoforms was up-regulated in the proximal intestine whereas no expression data were reported for the distal intestine (Tipsmark et al., 2010b). This indicates increased importance of the above mentioned AQPs in intestinal fluid absorption in a hyperosmotic environment. Indeed, the expression of AQP-1aa, -1ab, and -8ab at the protein level have been verified in SW acclimated Atlantic salmon by immunostaining by Madsen and co-workers (2011), whereas no protein expression patterns are available for salmon in FW or during smoltification. Although the protein abundance and cellular distribution has yet to be described during smoltification and compared between FW and SW, AQP-1aa and AQP-1ab in SW can be located to the brush border and sub-apical region of pyloric caeca, proximal and distal intestine membrane (Madsen et al., 2011). AQP-8ab was localized to the same area as 1aa and -1ab but in addition it was found also in the lateral regions of the enterocytes (Madsen et al., 2011). Moreover, a functional importance of AQPs in intestinal fluid absorption was suggested after a >50% reduction of intestinal fluid transport in non-everted gut-sac preparations treated with HgCl_2_, a potent AQP inhibitor (Madsen et al., 2011). Convincing data for the predominance of a transcellular route for intestinal fluid absorption has recently been shown in another euryhaline teleost, the killifish (*Fundulus heteroclitus*). In this species, osmotic clamping conditions increased the net mucosal to serosal water flux 10-fold, whereas the flux of different sized polyethylene glycols (PEG; 400, 900, and 4000) was unaffected (Wood and Grosell, 2012). Under these conditions, addition of HgCl_2_ reduced the fluid absorption by 60%, whereas the PEG permeability was increased 6–8 times. Thus, this study concludes that water and PEGs uses separate pathways to transfer across the intestinal epithelia and that the fluid absorption mainly uses a transcellular route, presumably through AQPs (Wood and Grosell, 2012). If the same relation can be observed in the intestine of salmonids remains to be determined.

An extensive amount of work is needed to fully elucidate the role of AQPs in intestinal fluid absorption of salmonids. Nevertheless, the expression of several AQPs at mRNA and protein level as well as decreased fluid absorption by HgCl_2_, clearly point toward a major importance of AQPs and the transcellular route for intestinal fluid absorption also in salmonids.

### The role of SGLT1

Significant volumes of water have been suggested to be transported via SGLT1 in the mammalian intestine (Loo et al., 2002). In rainbow trout, glucose homeostasis appears to be dependent on intestinal absorption and the presence of SGLT1 in the enterocytes has been verified at both mRNA and protein level (Polakof et al., 2010). Transport kinetics of glucose in the intestine of Atlantic salmon show highest transport rate in the pyloric caeca, intermediate in the proximal intestine and low in the distal intestine (Bakke-McKellep et al., 2000) which correlates well to the mRNA expression of SGLT1 in rainbow trout (Madsen et al., 2011). If the salmonid SGLT1 is involved in water flux in a similar manner as described for mammals, a regional difference in the contribution of SGLT1 to water transport would be expected. Interestingly, SGLT1 also appears to have a role in intestinal water transport of rainbow trout as Madsen et al. (2011) showed that the water transport could be reduced by 20% by blocking the SGLT1 transport with phlorizin. However, gut sac preparations from the whole intestinal tract, proximal and distal intestine together, were used in this study. Thus, no differentiation between intestinal regions was possible. High fluid uptake has been observed in pyloric caeca of chinook salmon (Veillette et al., 2005). It can be speculated that SGLT1 may be a major contributor to water absorption in this region as well as in the proximal intestine.

## Conclusions and future perspectives

**Figure 7:**
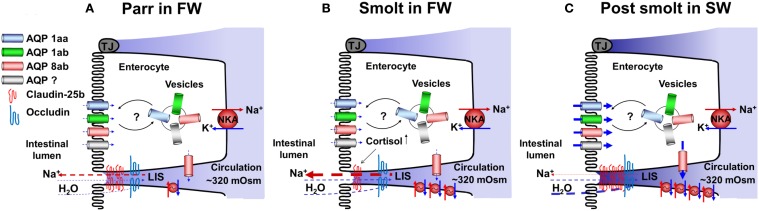
**A tentative model showing possible roles and positions of aquaporins (AQPs) and claudins in FW parr (A), during smoltification (B), and after acclimation to SW (C)**. In SW post-smolts, protein expression of AQPs 1aa, 1ab, and 8ab are present in the apical membrane, AQPs 1aa and 1ab in vesicles and AQP 8ab in the basolateral membrane **(C)**, with basis in these experiments and results of transcriptome analyses possible AQP positions in FW parr and smolts are suggested (**A** and **B**). Regarding tight junction proteins no protein expression has, as far as we know, been demonstrated. Thus the present model is based on transcriptome analyses of different claudin and occluding genes. NKA; Na^+^, K^+^-ATPase, LIS; lateral intercellular space.

The life cycle of anadromous salmonids makes these fish interesting to study as they are able to acclimate to both hypo- and hyperosmotic environments. In FW, smoltification prepares the hyperosmoregulatory parr for a life as a hypoosmoregulatory smolt in SW by increasing the drinking rate, intestinal NKA activity and ion co-transports and subsequently fluid transport (Figure 7). These changes are mediated, to a large extent, by the developmental increase in circulating plasma cortisol levels. Even though some important transporters behind the intestinal fluid absorption has been characterized and localized, expression at both mRNA and protein level of others, such as NKCC2, NHEs, SLC26A6, NCC, and NBC1 remains to be determined during the smoltification. The developmental elevation in plasma cortisol levels further results in increased paracellular permeability, probably through a down regulation of the barrier building claudin-25b in the intestine (Tipsmark et al., 2010a). In order to understand how the different isoforms of fish claudins modulate the intestinal permeability, functional studies needs to be assessed for each isoform to assign different claudins barrier building and/or pore forming characteristics. Recently, an intestinal epithelial cell line from the distal region, exhibiting epithelial-like structure, has been developed from rainbow trout (Kawano et al., 2011). If this cell line is suitable for Ussing chamber studies, over expression of the different claudins followed by monitoring of TER and P_app_ will provide information on how these proteins regulate the charge and size selectivity within the TJ. Nevertheless, the increased paracellular permeability observed during smoltification in FW, is suggested to result in an increased leakage of positive ions from the LIS back to the intestinal lumen and thus prevent the buildup of the osmotic ion gradient in the LIS essential for the SW adaptive fluid absorption. Thus, the increased paracellular permeability in FW allows for a preparatory increase of ion transporting activities (i.e., increased NKA activity) in the enterocytes without creating an efficient fluid absorption while the fish is still in FW. After transition to SW, the drinking rates, intestinal NKA activity and fluid absorption are maintained high. In parallel, the paracellular permeability decreases, probably due to up-regulation of claudin-25b (Tipsmark et al., 2010a). This decreases the leakage of ions into the lumen which in turn allows for the buildup of the fluid driving osmotic gradient in the LIS. The tightening of the paracellular permeability further suggests that the water flow is redirected from a paracellular route, to a more transcellular route, which can be accomplished either through the lipid bilayer and/or by incorporation and/or trafficking of AQPs into the intestinal epithelium. The relative importance of transcellular water permeability through the enterocyte lipid bilayer should be further investigated using polar lipid vesicles derived from intestinal enterocyte membranes of FW and SW acclimated salmonids, respectively. The protein expression of AQPs at the enterocyte cellular and subcellular levels should be investigated during smoltification and after SW acclimation. Moreover, crystallization studies can be used to dissolve the high resolution structures of AQPs. This information can in turn be used to study the function and regulation of AQPs in artificial liposomes where different extracellular conditions can be mimicked. These detailed mechanistic, molecular approaches should be complemented by functional studies using pharmacological tools for trans- and paracellular transporters and pathways respectively, while simultaneously monitoring fluid fluxes. This approach would provide important information on the relative importance of the paracellular and the transcellular pathway in salmonid fluid absorption in FW, during smoltification and after SW acclimation.

### Conflict of interest statement

The authors declare that the research was conducted in the absence of any commercial or financial relationships that could be construed as a potential conflict of interest.

## References

[B1] Ahrne S. Johansson Hagslätt M. L. (2011). Effect of lactobacilli on paracellular permeability in the gut. Nutrients 3, 104–117 10.3390/nu3010104 22254077 PMC3257727

[B2] Alves P. Soveral G. Macey R. I. Moura T. F. (1999). Kinetics of water transport in eel intestinal vesicles. J. Membr. Biol. 171, 177–182 10.1007/s002329900569 10489429

[B3] Amasheh S. Fromm M. Günzel D. (2011). Claudins of intestine and nephron - a correlation of molecular tight junction structure and barrier function. Acta Physiol. (Oxf.) 201, 133–140 10.1111/j.1748-1716.2010.02148.x 20518752

[B4] Anderson J. M. Van Itallie C. M. Fanning A. S. (2004). Setting up a selective barrier at the apical junction complex. Curr. Opin. Cell Biol. 16, 140–145 10.1016/j.ceb.2004.01.005 15196556

[B5] Ando M. (1975). Intestinal water transport and chloride pump in relation to sea-water adaptation of eel, *Anguilla japonica*. Comp. Biochem. Physiol. A Comp. Physiol. 52, 229–233 240556 10.1016/s0300-9629(75)80158-7

[B6] Ando M. Urita S. Nagahama H. (1975). Active transport of chloride in eel intestine with special reference to sea water adaptation. Comp. Biochem. Physiol. A Comp. Physiol. 51, 27–32 236883 10.1016/0300-9629(75)90408-9

[B7] Artursson P. Ungell A. L. Löfroth J. E. (1993). Selective paracellular permeability in two models of intestinal absorption: cultured monolayers of human intestinal epithelial cells and rat intestinal segments. Pharm. Res. 10, 1123–1129 10.1023/A:1018903931777 8415396

[B8] Bagnat M. Cheung I. D. Mostov K. E. Stainier D. Y. R. (2007). Genetic control of single lumen formation in the zebrafish gut. Nat. Cell Biol. 9, 954–960 10.1038/ncb1621 17632505

[B9] Bakke-McKellep A. M. Nordrum S. Krogdahl Å. Buddington R. K. (2000). Absorption of glucose, amino acids, and dipeptides by the intestines of Atlantic salmon (*Salmo salar* L.). Fish Physiol. Biochem. 22, 33–44

[B10] Bisbal G. A. Specker J. L. (1991). Cortisol stimulates hypo-osmoregulatory ability in Atlantic salmon, *Salmo salar* L. J. Fish Biol. 39, 421–432

[B11] Bjarnason I. MacPherson A. Hollander D. (1995). Intestinal permeability: an overview. Gastroenterology 108, 1566–1581 7729650 10.1016/0016-5085(95)90708-4

[B12] Blikslager A. T. Moeser A. J. Gookin J. L. Jones S. L. Odle J. (2007). Restoration of barrier function in injured intestinal mucosa. Physiol. Rev. 87, 545–564 10.1152/physrev.00012.2006 17429041

[B13] Brasitus T. A. Dudeja P. K. Worman H. J. Foster E. S. (1986). The lipid fluidity of rat colonic brush-border membrane vesicles modulates Na^+^-H^+^ exchange and osmotic water permeability. Biochim. Biophys. Acta 855, 16–24 10.1016/0005-2736(86)90183-5 3002472

[B14] Cahu C. L. Infante J. L. Z. Corraze G. Coves D. (2000). Dietary lipid level affects fatty acid composition and hydrolase activities of intestinal brush border membrane in seabass. Fish Physiol. Biochem. 23, 165–172

[B15] Cerda J. Finn R. N. (2010). Piscine aquaporins: an overview of recent advances. J. Exp. Zool. A Ecol. Genet. Physiol. 313, 623–650 10.1002/jez.634 20717996

[B16] Clarke L. L. (2009). A guide to Ussing chamber studies of mouse intestine. Am. J. Physiol. Gastrointest. Liver Physiol. 296, G1151–G1166 10.1152/ajpgi.90649.2008 19342508 PMC2697950

[B17] Colin D. A. Nonnotte G. Leray C. Nonnotte L. (1985). Na-transport and enzyme-activities in the intestine of the fresh-water and sea-water adapted trout (*Salmo gairdneri* R). Comp. Biochem. Physiol. A Comp. Physiol. 81, 695–698 2863061 10.1016/0300-9629(85)91049-7

[B18] Collie N. L. (1985). Intestinal nutrient transport in coho salmon (*Oncorhynchus kisutch*) and the effects of development, starvation, and seawater adaptation. J. Comp. Physiol. B 156, 163–174

[B19] Collie N. L. Bern H. A. (1982). Changes in intestinal fluid transport associated with smoltification and sea-water adaptation in Coho salmon, *Oncorhynchus kisutch* (Walbaum). J. Fish Biol. 21, 337–348

[B20] Cornell S. C. Portesi D. M. Veillette P. A. Sundell K. Specker J. L. (1994). Cortisol stimulates intestinal fluid uptake in Atlantic salmon (*Salmo salar*) in the post-smolt stage. Fish Physiol. Biochem. 13, 183–190 10.1007/BF00004356 24198188

[B21] Cummins P. M. (2012). Occludin: one protein, many forms. Mol. Cell. Biol. 32, 242–250 10.1128/MCB.06029-11 22083955 PMC3255790

[B22] Cutler C. P. Martinez A. S. Cramb G. (2007). The role of aquaporin-3 in teleost fish. Comp. Biochem. Physiol. A Mol. Integr. Physiol. 148, 82–91 10.1016/j.cbpa.2006.09.022 17126580

[B23] Daugherty A. L. Mrsny R. J. (1999). Regulation of the intestinal epithelial paracellular barrier. Pharm. Sci. Technolo. Today 2, 281–287 10407391 10.1016/s1461-5347(99)00170-4

[B24] Engelund M. B. Yu A. S. Li J. Madsen S. S. Færgeman N. J. Tipsmark C. K. (2012). Functional characterization and localization of a gill-specific claudin isoform in Atlantic salmon. Am. J. Physiol. Regul. Integr. Comp. Physiol. 302, R300–R311 10.1152/ajpregu.00286.2011 21975646 PMC3349389

[B25] Evans D. H. (2008). Teleost fish osmoregulation: what have we learned since August Krogh, Homer Smith, and Ancel Keys. Am. J. Physiol. Regul. Integr. Comp. Physiol. 295, R704–R713 10.1152/ajpregu.90337.2008 18525009

[B26] Field M. Karnaky K. J. Jr. Smith P. L. Bolton J. E. Kinter W. B. (1978). Ion transport across the isolated intestinal mucosa of the winter flounder, *Pseudopleuronectes americanus*. I. Functional and structural properties of cellular and paracellular pathways for Na and Cl. J. Membr. Biol. 41, 265–293 671526 10.1007/BF01870433

[B27] Fischbarg J. (2010). Fluid transport across leaky epithelia: central role of the tight junction and supporting role of aquaporins. Physiol. Rev. 90, 1271–1290 10.1152/physrev.00025.2009 20959616

[B28] Frizzell R. A. Halm D. R. Musch M. W. Stewart C. P. Field M. (1984). Potassium transport by flounder intestinal mucosa. Am. J. Physiol. 246, F946–F951 6742138 10.1152/ajprenal.1984.246.6.F946

[B29] Frizzell R. A. Smith P. L. Vosburgh E. Field M. (1979). Coupled sodium-chloride influx across brush border of flounder intestine. J. Membr. Biol. 46, 27–39 221659 10.1007/BF01959973

[B30] Fuentes J. Bury N. R. Carroll S. Eddy F. B. (1996). Drinking in Atlantic salmon presmolts (*Salmo salar* L.) and juvenile rainbow trout (*Oncorhynchus mykiss* Walbaum) in response to cortisol and sea water challenge. Aquaculture 141, 129–137

[B31] Genz J. Esbaugh A. J. Grosell M. (2011). Intestinal transport following transfer to increased salinity in an anadromous fish (*Oncorhynchus mykiss*). Comp. Biochem. Physiol. A Mol. Integr. Physiol. 159, 150–158 10.1016/j.cbpa.2011.02.011 21349342

[B32] Grass G. M. Sweetana S. A. (1988). *In vitro* measurement of gastrointestinal tissue permeability using a new diffusion cell. Pharm. Res. 5, 372–376 10.1023/A:1015911712079 3244649

[B33] Grosell M. (2010). The role of the gastrointestinal tract in salt and water balance, in Fish Physiology, eds Grosell M. Farrell A. P. Colin J. B. (San Diego, CA: Academic Press), 135–164

[B34] Grosell M. (2011). Intestinal anion exchange in marine teleosts is involved in osmoregulation and contributes to the oceanic inorganic carbon cycle. Acta Physiol. (Oxf.) 202, 421–434 10.1111/j.1748-1716.2010.02241.x 21362153

[B35] Grosell M. Genz J. Taylor J. R. Perry S. F. Gilmour K. M. (2009). The involvement of H+-ATPase and carbonic anhydrase in intestinal HCO3- secretion in seawater-acclimated rainbow trout. J. Exp. Biol. 212, 1940–1948 10.1242/jeb.026856 19483012

[B36] Grosell M. Gilmour K. M. Perry S. F. (2007). Intestinal carbonic anhydrase, bicarbonate, and proton carriers play a role in the acclimation of rainbow trout to seawater. Am. J. Physiol. Regul. Integr. Comp. Physiol. 293, R2099–R2111 10.1152/ajpregu.00156.2007 17761514

[B37] Haines T. H. (1994). Water transport across biological membranes. FEBS Lett. 346, 115–122 10.1016/0014-5793(94)00470-6 8206149

[B38] Halm D. R. Krasny E. J. Frizzell R. A. (1985a). Electrophysiology of flounder intestinal-mucosa. 1. Conductance properties of the cellular and paracellular pathways. J. Gen. Physiol. 85, 843–864 2410537 10.1085/jgp.85.6.843 PMC2215783

[B39] Halm D. R. Krasny E. J. Frizzell R. A. (1985b). Electrophysiology of flounder intestinal mucosa.2. Relation of the electrical potential profile to coupled NaCl absorption. J. Gen. Physiol. 85, 865–883 2410538 10.1085/jgp.85.6.865 PMC2215785

[B40] Hill A. E. Shachar-Hill B. Shachar-Hill Y. (2004). What are aquaporins for? J. Membr. Biol. 197, 1–32 10.1007/s00232-003-0639-6 15014915

[B41] Hill W. G. Rivers R. L. Zeidel M. L. (1999). Role of leaflet asymmetry in the permeability of model biological membranes to protons, solutes, and gases. J. Gen. Physiol. 114, 405–414 10.1085/jgp.114.3.405 10469730 PMC2229456

[B42] Houpe K. L. Malo C. Buddington R. K. (1997). Dietary lipid and intestinal brush border membrane phospholipid fatty acid composition and glucose transport of channel catfish. Physiol. Zool. 70, 230–236 9231396 10.1086/639584

[B43] House C. R. Green K. (1965). Ion and water transport in isolated intestine of the marine teleost, *Cottus scorpius*. J. Exp. Biol. 42, 177–189 14293034 10.1242/jeb.42.1.177

[B44] Huang K. C. Holt J. P. J. (1974). Ion transport and permeability studies on the intestine of Fundulus heteroclitus. Bull. Mt. Desert Is. Biol. Lab. 14, 39–41

[B45] Jutfelt F. Olsen R. E. Björnsson B. T. Sundell K. (2007). Parr-smolt transformation and dietary vegetable lipids affect intestinal nutrient uptake, barrier function and plasma cortisol levels in Atlantic salmon. Aquaculture 273, 298–311

[B46] Jutfelt F. Olsen R. E. Glette J. Ringø E. Sundell K. (2006). Translocation of viable *Aeromonas salmonicida* across the intestine of rainbow trout, *Oncorhynchus mykiss* (Walbaum). J. Fish Dis. 29, 255–262 10.1111/j.1365-2761.2006.00715.x 16677315

[B47] Jutfelt F. Sundh H. Glette J. Mellander L. Björnsson B. T. Sundell K. (2008). The involvement of *Aeromonas salmonicida* virulence factors in bacterial translocation across the rainbow trout, *Oncorhynchus mykiss* (Walbaum), intestine. J. Fish Dis. 31, 141–151 10.1111/j.1365-2761.2007.00879.x 18234022

[B48] Kawano A. Haiduk C. Schirmer K. Hanner R. Lee L. E. J. Dixon B. (2011). Development of a rainbow trout intestinal epithelial cell line and its response to lipopolysaccharide. Aquacult. Nutr. 17, E241–E252

[B49] Kerstetter T. H. White R. J. (1994). Changes in intestinal water absorption in Coho salmon during short-term seawater adaptation - a developmental study. Aquaculture 121, 171–180

[B50] Laforenza U. (2012). Water channel proteins in the gastrointestinal tract. Mol. Aspects Med. 33, 642–650 10.1016/j.mam.2012.03.001 22465691

[B51] Lande M. B. Donovan J. M. Zeidel M. L. (1995). The relationship between membrane fluidity and permeabilities to water, solutes, ammonia, and protons. J. Gen. Physiol. 106, 67–84 7494139 10.1085/jgp.106.1.67 PMC2229255

[B52] Leray C. Chapelle S. Duportail G. Florentz A. (1984). Changes in fluidity and 22, 6(n - 3) content in phospholipids of trout intestinal brush-border membrane as related to environmental salinity. BBA-Biomembranes 778, 233–238

[B53] Leung D. W. Loo D. D. F. Hirayama B. A. Zeuthen T. Wright E. M. (2000). Urea transport by cotransporters. J. Physiol. 528, 251–257 10.1111/j.1469-7793.2000.00251.x 11034615 PMC2270130

[B54] Li H. O. Yamada J. (1992). Changes of the fatty acid composition in smolts of masu salmon (*Oncorhynchus masou*), associated with desmoltification and sea-water transfer. Comp. Biochem. Physiol. A Physiol. 103, 221–226

[B55] Loh Y. H. Christoffels A. Brenner S. Hunziker W. Venkatesh B. (2004). Extensive expansion of the claudin gene family in the teleost fish, *Fugu rubripes*. Genome Res. 14, 1248–1257 10.1101/gr.2400004 15197168 PMC442139

[B56] Loo D. D. Wright E. M. Zeuthen T. (2002). Water pumps. J. Physiol. 542, 53–60 10.1113/jphysiol.2002.018713 12096049 PMC2290409

[B57] Loretz C. A. (1995). Electrophysiology of ion transport in teleost intestinal cells, in Fish Physiology, eds Chris M. W. Trevor J. S. (San Diego, CA: Academic Press), 25–56

[B58] Ma T. Verkman A. S. (1999). Aquaporin water channels in gastrointestinal physiology. J. Physiol. 517, 317–326 10.1111/j.1469-7793.1999.0317t.x 10332084 PMC2269340

[B59] Madara J. L. Pappenheimer J. R. (1987). Structural basis for physiological regulation of paracellular pathways in intestinal epithelia. J. Membr. Biol. 100, 149–164 3430571 10.1007/BF02209147

[B60] Madsen S. S. (1990). Cortisol treatment improves the development of hypoosmoregulatory mechanisms in the euryhaline rainbow trout, *Salmo gairdneri*. Fish Physiol. Biochem. 8, 45–52 24221896 10.1007/BF00004430

[B61] Madsen S. S. Olesen J. H. Bedal K. Engelund M. B. Velasco-Santamaria Y. M. Tipsmark C. K. (2011). Functional characterization of water transport and cellular localization of three aquaporin paralogs in the salmonid intestine. Front. Physiol. 2:56 10.3389/fphys.2011.00056 21941512 PMC3171111

[B62] Marshall W. S. Grosell M. (2005). Ion transport, osmoregulation and acid-base balance, in The Physiology of Fishes, 3rd Edn eds Evans D. H. Claiborne J. B. (Boca Raton, FL: CRC Taylor and Francis), 177–230

[B63] Matsuzaki T. Tajika Y. Ablimit A. Aoki T. Hagiwara H. Takata K. (2004). Aquaporins in the digestive system. Med. Electron Microsc. 37, 71–80 10.1007/s00795-004-0246-3 15221647

[B64] McCormick S. D. Hansen L. P. Quinn T. P. Saunders R. L. (1998). Movement, migration, and smolting of Atlantic salmon (*Salmo salar*). Can. J. Fish. Aquat. Sci. 55, 77–92

[B65] Mistry A. C. Chen G. Kato A. Nag K. Sands J. M. Hirose S. (2005). A novel type of urea transporter, UT-C, is highly expressed in proximal tubule of seawater eel kidney. Am. J. Physiol. Renal Physiol. 288, F455–F465 10.1152/ajprenal.00296.2004 15383403

[B66] Moeser A. J. Ryan K. A. Nighot P. K. Blikslager A. T. (2007). Gastrointestinal dysfunction induced by early weaning is attenuated by delayed weaning and mast cell blockade in pigs. Am. J. Physiol. Gastrointest. Liver Physiol. 293, G413–G421 10.1152/ajpgi.00304.2006 17525151

[B67] Musch M. W. Orellana S. A. Kimberg L. S. Field M. Halm D. R. Krasny E. J. (1982). Na^+^ -K^+^ -Cl^−^ co-transport in the intestine of a marine teleost. Nature 300, 351–353 7144890 10.1038/300351a0

[B68] Nedvetsky P. I. Tamma G. Beulshausen S. Valenti G. Rosenthal W. Klussmann E. (2009). Regulation of aquaporin-2 trafficking. Handb. Exp. Pharmacol. 133–157 19096775 10.1007/978-3-540-79885-9_6

[B69] Nielsen C. Madsen S. S. Björnsson B. T. (1999). Changes in branchial and intestinal osmoregulatory mechanisms and growth hormone levels during smolting in hatchery-reared and wild brown trout. J. Fish Biol. 54, 799–818

[B70] Oxley A. Jutfelt F. Sundell K. Olsen R. E. (2007). Sn-2-monoacylglycerol, not glycerol, is preferentially utilised for triacylglycerol and phosphatidylcholine biosynthesis in Atlantic salmon (*Salmo salar* L.) intestine. Comp. Biochem. Physiol. B Biochem. Mol. Biol. 146, 115–123 10.1016/j.cbpb.2006.09.007 17126582

[B71] Perrott M. N. Grierson C. E. Hazon N. Balment R. J. (1992). Drinking behavior in sea-water and fresh-water teleosts, the role of the renin-angiotensin system. Fish Physiol. Biochem. 10, 161–168 10.1007/BF00004527 24214213

[B72] Polakof S. Alvarez R. Soengas J. L. (2010). Gut glucose metabolism in rainbow trout: implications in glucose homeostasis and glucosensing capacity. Am. J. Physiol. Regul. Integr. Comp. Physiol. 299, R19–R32 10.1152/ajpregu.00005.2010 20357022

[B73] Powell D. W. (1981). Barrier function of epithelia. Am. J. Physiol. 241, G275–G288 7032321 10.1152/ajpgi.1981.241.4.G275

[B74] Preston G. M. Carroll T. P. Guggino W. B. Agre P. (1992). Appearance of water channels in Xenopus oocytes expressing red cell CHIP28 protein. Science 256, 385–387 10.1126/science.256.5055.385 1373524

[B75] Rey P. Rozas G. Andres M. D. Aldegunde M. Rebolledo E. (1991). Intestinal ATPases activities in domesticated rainbow trout (*Salmo gairdneri*) at different times of the year. J. Interdiscipl. Cycle. Res. 22, 261–270

[B76] Rosenthal R. Milatz S. Krug S. M. Oelrich B. Schulzke J. D. Amasheh S. (2010). Claudin-2, a component of the tight junction, forms a paracellular water channel. J. Cell Sci. 123, 1913–1921 10.1242/jcs.060665 20460438

[B77] Ruyter B. Moya-Falcón C. Rosenlund G. Vegusdal A. (2006). Fat content and morphology of liver and intestine of Atlantic salmon (*Salmo salar*): effects of temperature and dietary soybean oil. Aquaculture 252, 441–452

[B78] Santos J. Benjamin M. Yang P. C. Prior T. Perdue M. H. (2000). Chronic stress impairs rat growth and jejunal epithelial barrier function: role of mast cells. Am. J. Physiol. Gastrointest. Liver Physiol. 278, G847–G854 10859213 10.1152/ajpgi.2000.278.6.G847

[B79] Santos J. Yang P. C. Söderholm J. D. Benjamin M. Perdue M. H. (2001). Role of mast cells in chronic stress induced colonic epithelial barrier dysfunction in the rat. Gut 48, 630–636 10.1136/gut.48.5.630 11302959 PMC1728291

[B80] Saunders P. R. Santos J. Hanssen N. P. M. Yates D. Groot J. A. Perdue M. H. (2002). Physical and psychological stress in rats enhances colonic epithelial permeability via peripheral CRH. Digest. Dis. Sci. 47, 208–215 10.1023/A:1013204612762 11852879

[B81] Schneeberger E. E. Lynch R. D. (2004). The tight junction: a multifunctional complex. Am. J. Physiol. Cell Physiol. 286, C1213–C1228 10.1152/ajpcell.00558.2003 15151915

[B82] Segner H. Sundh H. Buchmann K. Douxfils J. Sundell K. Mathieu C. (2012). Health of farmed fish: its relation to fish welfare and its utility as welfare indicator. Fish Physiol. Biochem. 38, 85–105 10.1007/s10695-011-9517-9 21681416

[B83] Seidelin M. Madsen S. S. Blenstrup H. Tipsmark C. K. (2000). Time-course changes in the expression of Na^+^, K^+^-ATPase in gills and pyloric caeca of brown trout (*Salmo trutta*) during acclimation to seawater. Physiol. Biochem. Zool. 73, 446–453 10.1086/317737 11009398

[B84] Seo P. R. Teksin Z. S. Kao J. P. Polli J. E. (2006). Lipid composition effect on permeability across PAMPA. Eur. J. Pharm. Sci. 29, 259–268 10.1016/j.ejps.2006.04.012 16781125

[B85] Shehadeh Z. H. Gordon M. S. (1969). The role of the intestine in salinity adaptation of the rainbow trout, *Salmo gairdneri*. Comp. Biochem. Physiol. 30, 397–418

[B86] Shrimpton J. M. McCormick S. D. (1998). Seasonal differences in plasma cortisol and gill corticosteroid receptors in upper and lower mode juvenile Atlantic salmon. Aquaculture 168, 205–219

[B87] Smith H. W. (1930). The Absorption and excretion of water and salts by marine teleosts. Am. J. Physiol. 93, 480–505

[B88] Specker J. L. (1982). Interrenal function and smoltification. Aquaculture 28, 59–66

[B89] Specker J. L. Portesi D. M. Cornell S. C. Veillette P. A. (1994). Methodology for implanting cortisol in Atlantic salmon and effects of chronically elevated cortisol on osmoregulatory physiology. Aquaculture 121, 181–193

[B90] Specker J. L. Schreck C. B. (1982). Changes in plasma corticosteroids during smoltification of Coho salmon, *Oncorhynchus kisutch*. Gen. Comp. Endocrinol. 46, 53–58 7060935 10.1016/0016-6480(82)90162-9

[B91] Stefansson S. O. Björnsson B. T. Sundell K. Nyhammer G. McCormick S. D. (2003). Physiological characteristics of wild Atlantic salmon post-smolts during estuarine and coastal migration. J. Fish Biol. 63, 942–955

[B92] Stubbs C. D. Smith A. D. (1984). The modification of mammalian membrane polyunsaturated fatty acid composition in relation to membrane fluidity and function. Biochim. Biophys. Acta 779, 89–137 10.1016/0304-4157(84)90005-4 6229284

[B93] Sundell K. Jutfelt F. Agustsson T. Olsen R. E. Sandblom E. Hansen T. (2003). Intestinal transport mechanisms and plasma cortisol levels during normal and out-of-season parr-smolt transformation of Atlantic salmon, Salmo salar. Aquaculture 222, 265–285

[B94] Sundh H. Calabrese S. Jutfelt F. Niklasson L. Olsen R. E. Sundell K. (2011). Translocation of infectious pancreatic necrosis virus across the intestinal epithelium of Atlantic salmon (*Salmo salar* L.). Aquaculture 321, 85–92

[B95] Sundh H. Kvamme B. O. Fridell F. Olsen R. E. Ellis T. Taranger G. L. (2010). Intestinal barrier function of Atlantic salmon (*Salmo salar* L.) post smolts is reduced by common sea cage environments and suggested as a possible physiological welfare indicator. BMC Physiol. 10, 22 10.1186/1472-6793-10-22 21062437 PMC2992494

[B96] Sundh H. Olsen R. E. Fridell F. Gadan K. Evensen Ø Glette J. (2009). The effect of hyperoxygenation and reduced flow in fresh water and subsequent infectious pancreatic necrosis virus challenge in sea water, on the intestinal barrier integrity in Atlantic salmon, *Salmo salar* L. J. Fish Dis. 32, 687–698 10.1111/j.1365-2761.2009.01047.x 19500205

[B97] Tipsmark C. K. (2008). Identification of FXYD protein genes in a teleost: tissue-specific expression and response to salinity change. Am. J. Physiol. Regul. Integr. Comp. Physiol. 294, R1367–R1378 10.1152/ajpregu.00454.2007 18256141

[B98] Tipsmark C. K. Kiilerich P. Nilsen T. O. Ebbesson L. O. E. Stefansson S. O. Madsen S. S. (2008). Branchial expression patterns of claudin isoforms in Atlantic salmon during seawater acclimation and smoltification. Am. J. Physiol. Regul. Integr. Comp. Physiol. 294, R1563–R1574 10.1152/ajpregu.00915.2007 18321951

[B99] Tipsmark C. K. Madsen S. S. (2007). Identification of multiple FXYD genes in a teleost fish: tissue-specific expression and effect of salinity. Comp. Biochem. Phys. A 148, S121

[B100] Tipsmark C. K. Madsen S. S. (2012). Tricellulin, occludin and claudin-3 expression in salmon intestine and kidney during salinity adaptation. Comp. Biochem. Physiol. A Mol. Integr. Physiol. 162, 378–385 10.1016/j.cbpa.2012.04.020 22561661

[B101] Tipsmark C. K. Sørensen K. J. Hulgard K. Madsen S. S. (2010a). Claudin-15 and-25b expression in the intestinal tract of Atlantic salmon in response to seawater acclimation, smoltification and hormone treatment. Comp. Biochem. Physiol. A Mol. Integr. Physiol. 155, 361–370 10.1016/j.cbpa.2009.11.025 19969100

[B102] Tipsmark C. K. Sørensen K. J. Madsen S. S. (2010b). Aquaporin expression dynamics in osmoregulatory tissues of Atlantic salmon during smoltification and seawater acclimation. J. Exp. Biol. 213, 368–379 10.1242/jeb.034785 20086120

[B103] Tresguerres M. Levin L. R. Buck J. Grosell M. (2010). Modulation of NaCl absorption by [HCO3-] in the marine teleost intestine is mediated by soluble adenylyl cyclase. Am. J. Physiol. Regul. Integr. Comp. Physiol. 299, R62–R71 10.1152/ajpregu.00761.2009 20410468 PMC2904142

[B104] Usher M. L. Talbot C. Eddy F. B. (1988). Drinking in Atlantic salmon smolts transferred to seawater and the relationship between drinking and feeding. Aquaculture 73, 237–246

[B105] Usher M. L. Talbott C. Eddy F. B. (1991). Intestinal water transport in juvenile atlantic salmon (*Salmo salar* L.) during smolting and following transfer to seawater. Comp. Biochem. Physiol. A Comp. Physiol. 100, 813–818 1685374 10.1016/0300-9629(91)90297-p

[B106] Ussing H. H. Zerahn K. (1951). Active transport of sodium as the source of electric current in the short-circuited isolated frog skin. Acta Physiol. Scand. 23, 110–127 14868510 10.1111/j.1748-1716.1951.tb00800.x

[B107] Walsh P. J. Heitz M. J. Campbell C. E. Cooper G. J. Medina M. Wang Y. S. (2000). Molecular characterization of a urea transporter in the gill of the gulf toadfish (*Opsanus beta*). J. Exp. Biol. 203, 2357–2364 10887074 10.1242/jeb.203.15.2357

[B108] Walz T. Fujiyoshi Y. Engel A. (2009). The AQP structure and functional implications. Handb. Exp. Pharmacol. 190, 31–56 10.1007/978-3-540-79885-9_2 19096771

[B109] Van Itallie C. M. Anderson J. M. (2006). Claudins and epithelial paracellular transport. Annu. Rev. Physiol. 68, 403–429 10.1146/annurev.physiol.68.040104.131404 16460278

[B110] Van Itallie C. M. Holmes J. Bridges A. Gookin J. L. Coccaro M. R. Proctor W. (2008). The density of small tight junction pores varies among cell types and is increased by expression of claudin-2. J. Cell Sci. 121, 298–305 10.1242/jcs.021485 18198187

[B111] Veillette P. A. Sundell K. Specker J. L. (1995). Cortisol mediates the increase in intestinal fluid absorption in Atlantic salmon during parr-smolt transformation. Gen. Comp. Endocrinol. 97, 250–258 10.1006/gcen.1995.1024 7622019

[B112] Veillette P. A. White R. J. Specker J. L. (1993). Changes in intestinal fluid transport in Atlantic salmon (*Salmo salar* L) during parr-smolt transformation. Fish Physiol. Biochem. 12, 193–202 10.1007/BF00004367 24202777

[B113] Veillette P. A. White R. J. Specker J. L. Young G. (2005). Osmoregulatory physiology of pyloric ceca: regulated and adaptive changes in chinook salmon. J. Exp. Zool. A Comp. Exp. Biol. 303, 608–613 10.1002/jez.a.173 15945075

[B114] Veillette P. A. Young G. (2004). Temporal changes in intestinal Na^+^, K+-ATPase activity and *in vitro* responsiveness to cortisol in juvenile chinook salmon. Comp. Biochem. Physiol. A Mol. Integr. Physiol. 138, 297–303 10.1016/j.cbpb.2004.04.007 15313483

[B115] Velin A. K. Ericson A. C. Braaf Y. Wallon C. Söderholm J. D. (2004). Increased antigen and bacterial uptake in follicle associated epithelium induced by chronic psychological stress in rats. Gut 53, 494–500 10.1136/gut.2003.028506 15016742 PMC1774000

[B116] Verkman A. S. (2009). Aquaporins: translating bench research to human disease. J. Exp. Biol. 212, 1707–1715 10.1242/jeb.024125 19448080 PMC2683014

[B117] Verkman A. S. (2012). Aquaporins in clinical medicine. Annu. Rev. Med. 63, 303–316 10.1146/annurev-med-043010-193843 22248325 PMC3319404

[B118] Vikström E. Bui L. Konradsson P. Magnusson K.-E. (2009). The junctional integrity of epithelial cells is modulated by *Pseudomonas aeruginosa* quorum sensing molecule through phosphorylation-dependent mechanisms. Exp. Cell Res. 315, 313–326 10.1016/j.yexcr.2008.10.044 19038248

[B119] Watanabe S. Mekuchi M. Ideuchi H. Kim Y. K. Kaneko T. (2011). Electroneutral cation-Cl–cotransporters NKCC2β and NCCβ expressed in the intestinal tract of Japanese eel *Anguilla japonica*. Comp. Biochem. Physiol. A Mol. Integr. Physiol. 159, 427–435 10.1016/j.cbpa.2011.04.009 21539929

[B120] Wikman-Larhed A. Artursson P. (1995). Co-cultures of human intestinal goblet (HT29-H) and absorptive (Caco-2) cells for studies of drug and peptide absorption. Eur. J. Pharm. Sci. 3, 171–183

[B121] Wilhelm S. W. Suttle C. A. (1999). Viruses and nutrient cycles in the sea. Bioscience 49, 781–788

[B122] Wood C. M. Grosell M. (2012). Independence of net water flux from paracellular permeability in the intestine of *Fundulus heteroclitus*, a euryhaline teleost. J. Exp. Biol. 215, 508–517 10.1242/jeb.060004 22246259

[B123] Young G. (1986). Cortisol secretion *in vitro* by the interrenal of coho salmon (*Oncorhynchus kisutch*) during smoltification relationship with plasma thyroxine and plasma cortisol. Gen. Comp. Endocrinol. 63, 191–200 10.1016/0016-6480(86)90156-5 3023179

